# Age at type 2 diabetes diagnosis among adults in Germany in 2014, 2019 and 2020

**DOI:** 10.1007/s00592-023-02162-4

**Published:** 2023-07-23

**Authors:** Wolfgang Rathmann, Esther Seidel-Jacobs, Ramona Hering, Mandy Schulz, Annika Hoyer, Thaddäus Tönnies

**Affiliations:** 1 Institute for Biometrics and Epidemiology, German Diabetes Center, Leibniz Center for Diabetes Research at Heinrich Heine University, Auf’m Hennekamp 65, 40225 Düsseldorf, Germany; 2 German Center for Diabetes Research, Partner Düsseldorf, Munich-Neuherberg, Germany; 3 Department of Data Science and Healthcare Analyses, Central Research Institute for Ambulatory Healthcare in Germany (Zi), Berlin, Germany; 4 Biostatistics and Medical Biometry, Medical School EWL, Bielefeld University, Bielefeld, Germany

Many diabetologists in Germany and other European countries have the impression that the age of newly diagnosed type 2 diabetes has dropped, due to an increasing diabetes incidence in younger age groups [1]. On the other hand, health care changes during the COVID-19 pandemic may have led to decreased type 2 diabetes diagnoses [1].

The aim was to estimate the sex-specific age at diagnosis of type 2 diabetes in adults in Germany in 2014, 2019 and 2020. Estimates in 2020 could be influenced by decreased health consultations due to the COVID-19 pandemic. In this cross-sectional study, we used nationwide claims data of outpatient diagnoses of all statutory health insured individuals with at least one contact to a physician in the respective year (N ≈ 50 million aged 20 years and over). Individuals with diabetes diagnoses during the previous 3 years were excluded. Incident type 2 diabetes was defined using ICD-10 codes (E11-E14; confirmed diagnoses). To reduce false positive diagnoses, there had to be ≥ 1 specific diabetes coding in one of three subsequent quarters. Incident type 2 diabetes and population at risk were aggregated in 5-year age groups (age 20–24 years to ≥ 95 years) [1]. Sex-specific mean age (standard deviation, SD) at type 2 diabetes diagnosis was estimated using the midpoints of 5-year age-groups. Type 2 diabetes incidences (per 1000 persons) were estimated in three age groups (20–39, 40–59, ≥ 60 years) as the ratio of the numbers of cases and persons at risk. Analyses were carried out using R, v. 4.1.3 (R Foundation for Statistical Computing, Vienna, Austria).

There were 477,532 persons with newly diagnosed type 2 diabetes in 2014 (2020: n = 422,756) (Table 1). Average age (SD) at diagnosis was 63.1 (14.4) years in 2014 (men: 61.8 (13.3) years; women: 64.4 (15.3) years). Mean age at type 2 diagnosis decreased from 2014 to 2019 in both sexes, with a further decrease in 2020 (Table 1). 5-year differences (2014–2019) were larger in women (− 1.4 years) than in men (− 1.0 years) and further enlarged in 2020.

**Table 1:** Mean age at type 2 diabetes diagnosis in adults (≥ 20 years of age): nationwide statutory health insurance data in Germany (2014; 2019; 2020)

	All	Males	Females
Population at risk (number of incident type 2 diabetes)			
2014	49,662,605 (477,532)	21,471,639 (237,887)	28,190,966 (239,645)
2019	50,950,493 (449,982)	22,577,164 (232,108)	28,373,329 (217,874)
2020	50,644,395 (422,756)	22,389,767 (218,382)	28,254,628 (204,374)
Mean age at diagnosis (SD)			
2014	63.1 (14.4)	61.8 (13.3)	64.4 (15.3)
2019	61.9 (14.7)	60.8 (13.6)	63.0 (15.7)
2020	61.7 (14.7)	60.7 (13.6)	62.8 (15.8)
Difference (2014–19); years (95%CI)	− 1.2 (− 1.3, − 1.1) [*p* < 0.001]	− 1.0 (− 1.0, − 0.9) [*p* < 0.001]	− 1.4 (− 1.5, − 1.3) [*p* < 0.001]
Difference (2014–20); years (95%CI)	− 1.4 (− 1.4, − 1.3) [*p* < 0.001]	− 1.1 (− 1.1, − 1.0) [*p* < 0.001]	− 1.6 (− 1.7, − 1.5) [*p* < 0.001]
Type 2 diabetes incidence: (per 1,000 persons; 95%CI)			
All ages (20 years and over)			
2014	9.6 (9.6,9.6)	11.1 (11,11.1)	8.5 (8.5,8.5)
2019	8.8 (8.8,8.9) [*p* < 0.001]	10.3 (10.2,10.3) [*p* < 0.001]	7.7 (7.6,7.7) [*p* < 0.001]
2020	8.3 (8.3,8.4) [*p* < 0.001]	9.8 (9.7,9.8) [*p* < 0.001]	7.2 (7.2,7.3) [*p* < 0.001]
Age 20–39 years			
2014	1.8 (1.7, 1.8)	1.6 (1.6, 1.6)	1.9 (1.8, 1.9)
2019	2.0 (2.0, 2.0) [*p* < 0.001]	1.9 (1.9, 1.9) [*p* < 0.001]	2.1 (2.1, 2.2) [*p* < 0.001]
2020	2.0 (2.0, 2.0) [*p* < 0.001]	1.9 (1.8, 1.9) [*p* < 0.001]	2.1 (2.1, 2.1) [*p* < 0.001]
Age 40–59 years			
2014	9.1 (9.1, 9.2)	11.9 (11.8, 11.9)	7.0 (6.9, 7.0)
2019	9.2 (9.1, 9.2) [*p* = 0.19]	11.9 (11.8, 12.0) [*p* = 0.45]	7.0 (6.9, 7.0) [*p* = 0.37]
2020	8.8 (8.7, 8.8) [*p* < 0.001]	11.3 (11.2, 11.4) [*p* < 0.001]	6.7 (6.7, 6.8) [*p* < 0.001]
Age ≥ 60 years			
2014	19.1 (19.1, 19.2)	21.9 (21.7, 22.0)	17.3 (17.2, 17.4)
2019	16.2 (16.2, 16.3) [*p* < 0.001]	19.3 (19.2, 19.4) [*p* < 0.001]	14.1 (14.1, 14.2) [*p* < 0.001]
2020	14.9 (14.9, 15.0) [*p* < 0.001]	17.9 (17.8, 18.0) [*p* < 0.001]	12.9 (12.9, 13.0) [*p* < 0.001]

*p*-values: Welch t-test for difference in mean age at diagnosis between in 2019 or 2020 compared to 2014; χ2-test for difference in incidence between in 2019 or 2020 compared to 2014

In the age group ≥ 60 years, type 2 diabetes incidence decreased from 2014 to 2019 (Table 1). In middle-aged persons (40–59 years) incidence rates were stable over this period. In people aged 20–39 years, type 2 diabetes incidence increased from 2014 to 2019. During the COVID-19 pandemic in 2020, incidences decreased in the middle-aged and older population compared to 2019, whereas no change was observed in the youngest age group (Table 1).

The novel study finding of this study is a decrease in age at type 2 diabetes diagnosis from 2014 to 2020. Age-specific type 2 diabetes incidence rates showed an opposing tendency, with a decrease in the age group ≥ 60 years and an increase in people aged 20–39 years. However, changes in the age at diagnoses are not necessarily caused by changes in incidence rates. Changes in the age distribution of the population at risk can also cause variation in age at diagnoses. Although mean age at diabetes diagnoses is relevant for clinical practice and prevention, it should not be used alone to evaluate epidemiology of type 2 diabetes.

Although the data provide information about the vast majority (90%) of the German population, they are not representative of patients with private health insurance, who account for about 10%. Furthermore, the study population only included patients, who had actually consulted a physician at least once a year. For a complete epidemiological investigation of secular trends in diabetes prevalence, undiagnosed cases must also be included, which were not available. Finally, the data depend on the coding behaviour of the physicians and medical staff.

The present study adds important data to the still limited epidemiological evidence for type 2 diabetes in Europe. Several factors associated with the development of type 2 diabetes are most likely underlying the observed changes in type 2 diabetes incidence. First, the current report of the German Nutrition Society shows an increased consumption of vegetables (tomatoes, carrots, pulses) [2]. Meat consumption remains high, but fortunately the consumption of red meat (pork) is decreasing [2]. Likewise, total alcohol consumption is decreasing, with beer showing the highest decline [2]. Coffee consumption, on the other hand, is on the rise. Furthermore, the prevalence of active smokers among women has fallen by almost 30% since 2003 (2003: 30%; 2015: 22%) [3]. In men, the prevalence decreased by 28% (2003: 39%; 2015: 28%) [3]. Finally, diabetes incidence inclined by 36% in Denmark after introducing HbA1c for diagnosis (2012), mainly by a reduction in the number of persons with acute hyperglycemia with an HbA1c below the threshold of 6.5% [4]. In Germany, HbA1c-based diagnosis may also have lowered diabetes detection rates.

On the other hand, the prevalence of obesity has hardly changed in the last decades. According to the German Nutrition Report, 21% of men and 19% of women are currently obese among seniors over 65 years of age [2]. In the 18–29 years age group, obesity prevalence increased from 5.5% to 9.7% in women and from 5.4% to 8.9% in men from 2010 to 2014/2015 [5]. In line with this finding, the present study found an increased diabetes incidence (age 20–39 years; 2014–2019).

The COVID-19 pandemic has changed health care utilization. A lower number of consultations could have deteriorated diabetes detection. Therefore, the number of persons with newly diagnosed diabetes in primary care may have decreased in 2020 compared to 2019. Thus, the decreasing case numbers of type 2 diabetes from 2019 to 2020 in our study are most likely due to a lower disease detection rate. Since the data only include information on the number of incident cases and the population at risk over a 1-year period, detailed analyses of the pandemic's impact on the number of healthcare contacts were not possible. Therefore, both changes in risk factors and health care influence diabetes incidence.

In conclusion, the present analysis of a large health insurance sample in Germany indicated a trend of a decreasing age at type 2 diabetes onset, which was further lowered during the COVID-19 pandemic.

## Data Availability

Due to data protection legislation in Germany statutory health insurance data on the study subjects cannot be released.
